# The emergence of community health worker programmes in the late apartheid era in South Africa: An historical analysis

**DOI:** 10.1016/j.socscimed.2010.06.009

**Published:** 2010-09

**Authors:** Nadja van Ginneken, Simon Lewin, Virginia Berridge

**Affiliations:** a Faculty of Public Health and Policy, London School of Hygiene and Tropical Medicine, Keppel St, London WC1E 7HT, UK; b Health Systems Research Unit, Medical Research Council of South Africa; c Norwegian Knowledge Centre for the Health Services; d Centre for History in Public Health, London School of Hygiene and Tropical Medicine, United Kingdom

**Keywords:** Community health workers, Community health worker (CHW) policy, South Africa, Oral history, Apartheid, Task-shifting, Community participation

## Abstract

There is re-emerging interest in community health workers (CHWs) as part of wider policies regarding task-shifting within human resources for health. This paper examines the history of CHW programmes established in South Africa in the later apartheid years (1970s–1994) – a time of innovative initiatives. After 1994, the new democratic government embraced primary healthcare (PHC), however CHW initiatives were not included in their health plan and most of these programmes subsequently collapsed. Since then a wide array of disease-focused CHW projects have emerged, particularly within HIV care.

Thirteen oral history interviews and eight witness seminars were conducted in South Africa in April 2008 with founders and CHWs from these earlier programmes. These data were triangulated with written primary sources and analysed using thematic content analysis. The study suggests that 1970s–1990s CHW programmes were seen as innovative, responsive, comprehensive and empowering for staff and communities, a focus which respondents felt was lost within current programmes. The growth of these earlier projects was underpinned by the struggle against apartheid. Respondents felt that the more technical focus of current CHW programmes under-utilise a valuable human resource which previously had a much wider social and health impact. These prior experiences and lessons learned could usefully inform policy-making frameworks for CHWs in South Africa today.

## Introduction

Community health workers (CHWs) are increasingly advocated as a potential solution to overcoming current shortfalls in human resources for health in different settings (Chopra, Munro, Lavis, Vist, & Bennett, 2008; Lewin et al., 2010; WHO, 2008). CHW is an umbrella term used for a heterogenous group of lay health workers. Their remit can range from implementing biomedical interventions to acting as community agents of social change (Lewin et al., 2010; Werner & Bower, 1982). This paper defines CHWs as people chosen within a community to perform functions related to healthcare delivery, who have no formal professional training or degree. CHWs initially gained global support at the 1978 Alma Ata conference on primary healthcare. They were seen as a key element of the strategy to achieve WHO’s goal, set in 1975, of ‘Health for All by the year 2000’. Many CHW programmes were established in the 1970s in low- and middle-income countries (Walt & Gilson, 1990). However, interest waned in the late 1980s and 1990s for several reasons: structural adjustment programmes; government failure in countries where large programmes were operational; and changes in ideology (Frankel, 1992; Walt & Gilson, 1990; WHO, 1986).

South Africa has a rich history of CHW projects that burgeoned during the repressive regime of apartheid (Table 1 juxtaposes key historical and project events). Under this racially and politically divided regime, healthcare was intentionally inequitably distributed (WHO, 1983). Among the first CHWs were malaria assistants trained in the late 1920s by G.A. Park Ross, a senior health officer in Natal and Zululand (MacKinnon, 2001). In the 1940s, despite an early Smuts government advocating racial segregation, supporters of social medicine initiated the ‘health centre’ movement. Chief among the politicians involved was Henry Gluckman, the then Minister of Health, who had been influenced by the United Kingdom’s Beveridge Report (1942). The ambitious 1942 National Health Service Commission and 1945 Gluckman Report set out to provide “unified healthcare to all sections of the people of South Africa”. They addressed both the social and biomedical causes of disease, responding in part to concerns regarding the effects of poor health on black migrant labourers’ and miners’ productivity (Phillips, 1993). However, the government only adopted the recommendation to establish health centres (Jeeves, 2000, 2005; Marks, 1997). Modelled on the rural health centre in Pholela (near Durban) initiated by Sidney and Emily Kark – a progressive and politically well connected medical couple – these centres were staffed by community nurses and assistants who treated and surveyed health problems (Kark, 1951; Tollman, 1994). Only 40 of the suggested 400 centres were eventually built to serve black communities. This service became racialised and gained a reputation for being a “second class service” (Marks, 1997).

As the Afrikaner National Party strengthened from 1948, the government withdrew its support from these centres. Many centres closed and a number of their founders, including the Karks, went into exile (Marks, 1997). From the 1960s to 1980s, when the bantustans (‘homelands’) became ‘independent’, the responsibility for the forcibly relocated black population’s health care was given to the ‘homeland’ governments (van Rensburg & Harrison, 1995). Most of these governments were under-resourced and corrupt and thus neglected health service funding.

Another phase of CHW projects began in the 1970s and continued into the 1990s, established mostly by individuals or small civic or religious organisations (Tollman & Pick, 2002). There was a growing conviction from the late 1980s that apartheid would soon end, particularly after African nationalist organisations were unbanned and their leaders released (Baldwin-Ragavan, Gruchy, & London, 1999). This encouraged progressive thinkers and academics to develop community initiatives and formulate advice which they hoped would inform a new government’s health policies (Price, 1993).

In 1994, South Africa welcomed its first democratic government. Though the government adopted the district health system (DHS) as the cornerstone of their national health plan, CHWs were not included. Subsequently, many CHW projects collapsed as international donors withdrew their earlier support, or redirected their funds through government departments. In recent years, an uncoordinated array of CHW programmes has re-emerged, mainly within healthcare for people living with HIV/AIDS (Friedman, 2005).

The early history of the 1930s and 1940s CHW projects has been analysed elsewhere (Jeeves, 2000, 2005; Marks, 1997; Tollman, 1994). However the late apartheid period (post-1970s) lacks historical analysis. This study aims to explore the factors affecting these late apartheid projects’ evolution. This historical analysis intends to contribute to current debates on the appropriateness, effectiveness and sustainability of CHW initiatives within South Africa and to similar global debates.

## Methods

Our approach used oral history interviews (in-depth, open-ended interviews seeking people’s reconstruction and interpretation of events) with founders, coordinators and health workers of CHW initiatives active during the study period. This approach was chosen as people working within CHW projects at this time were busy ‘doing’ rather than ‘documenting’. This technique recognises individual experience, often missing from standard social histories, and gathers unrecorded information (Perks, 1992).

To select interviewees, four contacts known to one of the authors (SL) helped identify further participants through snowballing. Participants were chosen from urban and rural projects where documentation was available. Of 54 potential interviewees, 39 were selected on the basis of representativeness of professional background, involvement in CHW programmes, and availability. Tragically, one key participant, Ivan Toms, died unexpectedly before his interview. A total of 38 participants were therefore interviewed from 10 projects (Table 2). One author (NvG) visited five provinces (Western Cape, Eastern Cape, Kwazulu-Natal, Mpumalanga and Limpopo) in South Africa in April 2008 to conduct 10 oral history interviews. An additional two interviews were conducted by phone, and one in London. Eight witness seminars (focus groups with two to seven participants from the same project) were also conducted, partly out of convenience but also because groups may encourage recollection of events. This research’s principal limitations are that snowballing may not have reached saturation and the chosen CHW initiatives may not have included the full range of contemporaneous programmes.

Most interviews were conducted in English; four required an interpreter (Zulu and Shangaan) and professional translation. Interviews explored how respondents became involved in CHW programmes, their experiences and their views on CHW programmes’ development. The interview guide was adapted to incorporate emerging themes.

Primary and secondary historical sources were obtained from libraries (UK and South Africa), government databases, the South African National Archives in Cape Town and from bibliographies. Grey literature held by interviewees (conference papers, reports, minutes, theses, photographs) was reviewed. The interviews were manually transcribed, coded and analysed by one author (NvG) using thematic content analysis. The other authors read selected transcripts and commented on emerging themes.

The analysis involved an inductive process to identify emerging themes. Constant comparison ensured that the themes reflected the original data. Oral sources were cross-examined with written material. This methodological triangulation allowed the identification of critical perspectives and emerging themes (Green & Thorogood, 2004).

Because some respondents requested anonymity, participants have been kept anonymous. Their quotes are coded according to participants’ professional background (C: coordinator; CHW: community health worker; D: doctor; N: nurse; F: founder). This research was approved by the ethics committees of the London School of Hygiene and Tropical Medicine and the University of Cape Town.

## Results

### How CHW programmes started

The driving force for non-governmental organisations’ (NGO) or rural health initiatives was often the desire of individuals to address the health of the under-served black majority. Most leaders of these initiatives were white doctors or nurses as oppression and poverty made it difficult for blacks to establish such infrastructures. Ithuseng health centre project set up by Mamphela Ramphele was one exception to this (Ramalepe, 1992). Involvement was sometimes fuelled by religious conviction (CHW12, C3) or by guilt about their privileged position compared to racially-oppressed black counterparts (C9).

Founders of many programmes explained that these projects arose during a time of growing discontent with apartheid, expressed through uprisings and demonstrations. The promise that the 1977 Public Health Act would expand healthcare for the black population remained unfulfilled (De Beer, 1984; Digby, 2006). The health and social problems experienced by the black majority worsened, as documented in the *Second Carnegie Inquiry into Poverty and Development in South Africa* (1984), to which some project founders contributed (Wilson & Ramphele, 1989).

During this period, CHW projects often started as single interventions to address what was seen as the greatest need (Table 2). The Elim Care Groups, spear-headed by the Swiss ophthalmologist Erika Sutter, responded to trachoma (an eye infection causing blindness). The Newlands and Chalumna projects, led by Trudy Thomas, a paediatrician, set up a nutrition scheme to respond to kwashiorkor (protein-energy malnutrition). The success of these single interventions led them later to address wider health issues in their communities.

Health projects, such as the Empilisweni SACLA (South African Christian Leadership Association) in an informal settlement outside Cape Town, and Health Care Trust’s (HCT) rural health initiative in Cala in the Transkei (now Eastern Cape) were motivated partly by community requests:

“*We weren’t looking for long term projects. We were approached to do these so I think it was something that we had as part of our values. We weren’t just ‘go and plonk ourselves’ in communities. It had to be something that we were approached by.”* (F7)

The motive for helping the black population was not always altruistic. There was also fear of a spill-over of ‘black diseases’ to the white community. This provided an incentive for a study on health and urbanisation to assess the impact of black urban migration on white city dwellers. Prevention strategies to ‘sanitise’ the most disadvantaged are globally recognised in history across public health reform (Pelling, Berridge, Harrison, & Weindling, 2001). This study ultimately led to the creation of the Centre for Epidemiological Research in Southern Africa (CERSA), which included progressive thinkers concerned with documenting and addressing the ill health of the underprivileged (F5).

The Karks’ Community Oriented Primary Care (COPC) model, developed in South Africa, contributed to shaping the 1977 Alma-Ata declaration and subsequent global community health movements. It also influenced later projects in South Africa. The Karks’ visits to Johannesburg and Durban in the 1980s and 1990s contributed to academics reviving surveillance/research-based projects based on the COPC approach. Mamre (in the Western Cape) and the Agincourt site (in Gazankulu, now Limpopo Province) of the University of Witwatersrand Health Systems Development Unit, developed and utilised participatory research approaches to create an important body of evidence on community health needs (Katzenellenbogen, Hoffman, & Miller, 1990; Tollman, 1999).

Leaders of non-academic civic projects drew less influence from the Karks’ model. Though South Africa did not attend the Alma Ata conference due to international sanctions, project founders embraced these principles as they reflected and justified their efforts. One founder explained why:

*“Immediately…I was taken up with the idea. In fact Alma-Ata was in 1978, so ideas about primary healthcare were floating around at that time and were starting to get formalised. What was clear to me was that [our project] had been practising PHC for nearly two decades before that. Because if you looked at what the principles of Alma-Ata were, things like community involvement, community development, appropriate health technologies, using a basic approach, even… basic equity. I mean there were things of course that weren*’*t being done, but some of those principles were being implemented and I felt very much at home. And for the next decade we really tried to make it a living example of primary healthcare in action.”* (F2)

Some respondents, particularly from repressive regime areas like the Ciskei, felt that their projects started in isolation and had few external influences as political sanctions hindered communication and access to information from outside of South Africa:

*“I was the only one. Mine was the only community health department. There weren*’*t any others in this province. There was no such thing as community health work…you know. I was just the clinic doctor and then the sense of a community health service grew.”* (F8)

In the late 1980s, conditions became more favourable to information exchange. Health activism grew alongside anti-apartheid activism. A network of local community health organisations formed the Progressive Primary Health Care Network (PPHCN). Supported by the National Medical and Dental Association and the (Kaiser-Foundation, 1988), it strengthened project cross-fertilisation and collaboration to formulate a future primary healthcare strategy (NPPHCN, 1986).

### The political nature of CHW projects

Most respondents felt that healthcare provision was inseparable from democracy, reflecting De Beer’s (1984) description of apartheid as the most important ‘disease’ affecting South Africa. Some respondents’ conviction that politics and health are connected explained their involvement in political activism. This put them at significant risk of detention without trial (D1, F3, F6), receiving threats (C11) or being harassed (CHW1), but did not hinder their commitment to work.

Other respondents were not politically active or found it too dangerous. They masked their desire for political change under the banner of healthcare provision while simultaneously challenging the status quo by empowering CHWs to become agents of social change. The Valley Trust, HCT and SACLA, for example, successfully introduced democratic community structures and elections within their projects. As one of the founders said:

*“I felt we needed to bring in the social aspects, where we needed to bring in elements of community involvement. Dangerous stuff at that time, because working with black communities was on the fringe of social revolution, but luckily primary healthcare permitted that ideology.”* (F2)

Most of the respondents who were active politically worked in areas where major political and social injustices had been carried out. The government’s systematic attempt in the 1980s to crack down on ‘illegal’ squatter areas through encouraging community riots led to a local SACLA clinic closing in 1986. Individuals working within projects that had some approval from their ‘homeland’ governments, such as Agincourt/Manguzi in Gazankulu and Valley Trust in former Kwazulu, were less likely to be heavily involved in political activism. A project coordinator felt that their work was part of the struggle for democracy.

*“During apartheid our main struggle was for freedom. Once that was achieved our remit was over.”* (C10)

This statement, which was reflected by many respondents, raises the question whether the same level of commitment of health workers to communities can be reproduced in a more democratic political climate in which human rights are less threatened.

### Innovative and experimental leadership, supervision and training

Respondents saw the presence of a charismatic idealistic leader, who had a firm development approach, as key to six projects’ success (Valley Trust, SACLA, HCT, Elim, Chalumna/Newlands and Rural Foundation). Ivan Toms, who helped establish the Empilisweni SACLA clinic, was seen as an example of such a leader and as crucial to the project’s success. In addition to actively defending the clinic and community during the mid 1980s’ riots, respondents described how he enlisted and trained lay people to work as CHWs or management staff, and empowered community members and staff to later adopt full managerial and clinical responsibility (C2, CHW1).

Respondents from all projects admitted to being experimental. Supervision, training and management of staff and CHWs were often done on an ad-hoc basis, as outlined by a SACLA doctor:

*“Those first CHWs were a huge experiment. We were just flying by the seat of our pants, we didn*’*t know what we were doing. We equipped them with basic medications and dressings and so on. And they were fantastic, so they were with the project for many, many years.”* (C3)

Project leaders were health professionals or academics with little experience in management – they were “trying things and seeing if they worked” (C10). Management difficulties sometimes developed, such as when SACLA and Rural Foundation became larger and more complex (C3, F10). One Elim report (*Annual report*, 1980) outlined difficulties of project expansion such as staff shortages and inadequate delegation. These caused management overload and demotivation of staff. Some projects, however, successfully involved communities. Brown’s Farm health-clinic lay managerial committees, and Elim and Rural Foundation coordinators were good mediators for enhancing community participation and dissipating personal and political disputes. However, community participation was never comprehensive – rather, it was restricted to certain tasks within projects. With the exceptions of SACLA and Elim, projects were established and run exclusively by people from outside the communities served.

Experienced programme clinicians and leaders developed and undertook hands-on supervision and ongoing training of CHWs and coordinators. Most CHWs described their supervision as informative and non-threatening:

*“On the farm [the supervisor] walk with me and the house-visit. And she look at us. And when something not right she don*’*t say*: *‘He he he, no’. She go with us in the clinic. In the container, we sit down, and she say: ‘Do you remember, what did you learn?’”* (CHW3)

### Appropriateness and adaptability

Adapting the project’s goals to community needs was important. Selina Maphorogo, the first CHW motivator (and later director) of the Elim Care Groups, re-shaped the project by adopting culturally-sensitive methods for delivering community health messages. These methods were reported as effective and sustained through the project’s history (Maphorogo, Sutter, & Jenkins, 2003).

Projects in their early stages, or which were geographically and ideologically isolated, were innovative in their use of appropriate technology and training approaches. Many succeeded despite sanctions in accessing international literature and low technology resources. They adapted key CHW training guides including the Chinese *Barefoot doctors manual* (Hunan-Zhong, 1977), Werner’s books *Where there is no doctor* (1977) and *Helping health workers learn* (1982) as well as WHO guidelines (1992). The Rural Foundation and Elim also used UNICEF tools such as Road-to-Health charts. In the late 1980s, networks wanted to create a feasible training model for the post-apartheid era. Emerging training centres (such as the PPHCN learning centre) were modelled, in part, on the Institute of Family and Community Health (IFCH) (1945–1961) which trained the 1940s’ health centres.

In the late 1980s, these projects also adapted to a changing disease burden in South Africa, moving away from child survival towards chronic diseases and HIV (Bradshaw et al., 1999). SACLA, Mamre and HSDU trained CHWs who specialised in rehabilitation, chronic disease and HIV. This also coincided with the move to a more selective PHC approach, influenced by international criticisms that the comprehensive PHC approach provided few concrete recommendations (Cueto, 2004).

There was an interesting paradox, which several key informants recognised. These CHW projects, they suggested, experienced a ‘golden’ era under the constraints of apartheid and lack of political freedoms. Projects were free to respond innovatively to needs. Funders – whether international donors (for most projects) or ‘homeland’ governments (as for Valley Trust) – had minimal requirements. Project leaders felt their impact was greatest on community health and development during apartheid. In contrast, they criticised current funding for being constrictive and conditional, and thus hindering creativity and local adaptation. However these divergent views may result from a tendency to romanticise the achievements possible in times of struggle and to resist, as many did globally, the emerging funding bureaucracy of the 1990s.

### Links with communities

In the late apartheid era, some local authorities felt threatened by the growing influence of projects (Toms, 1987). Also communities sometimes found it difficult to accept CHW programmes. With individuals expressing jealousy regarding CHWs’ status (C6, F10, CHW-workshop, 1982). In addition, local expectations were hard to meet. For example, within the HCT-Cala project, villagers “did not get involved unless remuneration for services or products was guaranteed” (Alperstein & Bunyonyo, 1998). Participation fluctuated and depended on social and power relationships, and satisfactory incentivising, as described in the wider literature (Frankel, 1992).

Despite these challenges, rural and peri-urban projects reported some success in retaining CHWs in voluntary or partially paid work and in community ownership of projects:

*“So we started January 1987… and we had patients that followed us from Old Crossroads. Because we moved to New Crossroads, that community welcomed us. So we had patients who followed us, the chronic patients saying that ‘we can*’*t do without you.’”* (C2)

With the shift to employment within the public health system following the democratic elections, many CHWs felt that their accountability to the community had changed. They were no longer flexible community-based workers, but located in health clinics full-time. A few missed community work intensely (CHW3, CHW6). However many CHWs now resented unpaid requests from fellow villagers:

*“They used to come to my house asking for help after even after working hours. I used to help them but now I am unable, I tell that I am tired as I have started working at 06h00 in the morning until 16h00 in the afternoon.”* (CHW7)

Coordinators (C11, C6) and founders (F2, F8) also commented on changes in CHWs’ attitudes since 1994:

*“There*’*s a very serious materialist dependency. I hate it but I have to face up that it has happened… It*’*s not that I am saying it was idealistic, the community health workers were at least as enthusiastic as I. …I can*’*t talk about the CHWs now in those glowing terms. The government now has this huge thing, they*’*ve got this small business programme – the pay roll. And the village health worker…, if the pay doesn*’*t come out, they ‘toy–toy’, they don*’*t go to work”* (F8)

There is likely to be an element of romanticising the past in describing volunteers as only committed during the apartheid era. However, introducing a stipend would expectedly reduce a volunteer’s willingness to work unpaid. The CHWs interviewed, who had worked in both the old and new systems, rejected volunteering. This is supported by contemporaneous literature (Binedell, 1990; HCT, 1982, pp. 45–50) and by recent findings in the Free State province (Schneider, Hlophe, & van Rensburg, 2008).

### CHWs within the health system

Whether CHWs can adequately bridge the gap between the formal health service and the community has long been debated internationally (Walt & Gilson, 1990). CHWs in Mamre and Elim reported that knowledge of their communities allowed them to successfully bridge the gap between researchers and communities. For example, they explained the purpose of community surveys in culturally-sensitive ways. But because some CHWs, for example in Elim, officially reported to the nurse-in-charge at the hospital, they did not bridge the gap with health services but were instead viewed as the lowest level of the health service hierarchy.

Many dedicated nurses (including five coordinators and three founders interviewed) played a significant role in supporting and training CHWs within NGO projects (Clarke, 1991; Mamre, 1992). Some CHW literature advocates nurses as ideally placed to support CHWs (Buch, Evian, Maswanganyi, Maluleke, & Waugh, 1984; Roscher, 1990). However some CHW respondents were disparaging of clinical supervision by hospital nurses, one commenting that it was only by “the will of God” that nothing disastrous happened during childbirth (CHW7). One founder suggested why nurses’ supervision was poor:

*“[The nurse facilitators] wouldn*’*t have that kind of vision or experience of working with communities in a democratic way, so they would tend to be bureaucratic and play things by the book. They could supervise but it was much more mechanical. And that I found that was not helpful in developing the analytic skills of the CHWs.”*(F2)

CHWs were not necessarily welcomed by formal health system staff. Although the South African Nursing Council – the main regulatory body for the profession – supported CHWs in principle (Marks, 1994), in practice nurses on the ground were reportedly intimidating and rude to them (C8). One doctor interviewed explains:

*“The attitude of the nurses is very, very problematic, they*’*re also so hierarchical now. My thesis is that nurses are fighting a feminist battle in their work place, black nurses in particular, because they have been so oppressed and the present health system oppresses them too, but there*’*s a bit of a perverse feminism acting there.”* (F8)

This quote illustrates that nurses have, in both the late apartheid and post-apartheid eras, enforced hierarchies within healthcare with CHWs in health centres often becoming nursing subordinates (Schneider et al., 2008). These limitations of nursing care in South Africa have been discussed extensively elsewhere (Marks, 1994; Stein, Lewin, & Fairall, 2007; Wood & Jewkes, 2006), with Marks (1994) noting that nurses may feel their role is threatened within primary healthcare, particularly in light of potentially professionalising CHWs.

### The end of an era: the closure of CHW programmes in the 1990s

Respondents noted that the early 1990s were a time of transition out of the bleak system of apartheid (F9) towards a more idealistic vision of the future (F8). Progressive community health leaders were active in formulating health policy which informed the ANC’s forthcoming national health plan (NPPHCN, 1992a, 1992b). Sidney Kark also held many meetings with health officials and academics to promote the Community Oriented Primary Care decentralised approach with strong community involvement (Kark, 1981).

Respondents were surprised that the new 1994 government had dropped support for CHW projects (*A national health plan for South Africa*, 1994), as the 1992 draft of the ANC health plan had favoured CHW coverage (Slabber, 1992). Despite decentralisation being a key element of the new health plan (van Rensburg, 2004) the incoming minister of health, Nkosasana Zuma, believed that CHWs would provide second rate care (F2). Rather, a professionalised team of doctors and nurses was visualised. In addition, health service leadership was preoccupied with structural transformation of the health sector (Tollman & Pick, 2002). Respondents felt the 1994 health plan was poorly informed with regards to CHWs:

*“[It was] highly ambitious, had little connection to and had not consulted contemporary projects on what they were actually doing and achieving.”* (F9)

Interviewees understood why international funders had redirected their support to the new democratic government. However they were frustrated that established community-based initiatives folded because of the government’s idealistic vision of a professional-driven DHS which, in their view, failed to incorporate adequately existing South African experiences. Some projects survived with meagre charitable contributions (Elim, SACLA). A few, such as the Valley Trust, continued to receive government support and helped to develop a provincial health plan.

The government’s decision in the mid-1990s to provide free primary healthcare was a further blow to organisations such as the Rural Foundation which had relied partly on community contributions. This decision changed community expectations of projects.

The HIV epidemic also negatively impacted several small vertical projects. Respondents suggested providing care to people living with AIDS in the pre-antiretroviral drugs era had diverted funds away from CHW projects. In the late 1990s, when PPHCN struggled for funds, a budget ten times its global budget was allocated to it to run the national AIDS programme (F2). The AIDS epidemic also shifted CHWs towards being single-purpose workers – a phenomenon also noted in Britain (Berridge, 1996). A comparison of past and present CHW programmes is shown in Table 3. Respondents saw these changes as having shattered the ideal of community-oriented and comprehensive primary care (Oppenheimer & Bayer, 2007).

### Has more recent community health policy been informed by the past?

The Community Oriented Primary Care approach informed the development of the DHS in the late 1990s. However, it has been noted that its community focus and epidemiological surveillance have not been implemented widely, mainly because of a poor management capacity and accountability to communities (Moosa, 2006; Tollman & Pick, 2002).

More recently, South Africa established a CHW Framework (2004) to guide the development of a national CHW programme. This aimed to establish cohesion between old and new CHW community-based organisations and to address the growing crisis of health worker shortage. Interviewees felt “this policy [had] come too late” (C10, F3) as this Framework had only drawn upon the newer single-purpose fragmented projects not upon apartheid era comprehensive community health initiatives. One interviewee, one of the few to be consulted prior to the finalisation of this policy, was disappointed:

*“The policy had ignored the recommendations we had made based on the spirit and the experience of the CHW projects [in the 1970–1990s]”* (C10)

This statement equates to what Lund (1993) called a policy paradox in describing the 1992 national CHW policy draft. The policy, she argues, attempts “to give the category of CHW a place, but whilst so doing, has failed to recognise diversity, needs and flexibility, thus invalidating its initial aim.”

Others suggested that implementation of the CHW Framework remains rigid and gives insufficient focus to rural areas (Friedman, 2005). The Framework has also been criticised for its vague and conflicting statements about remuneration and responsibility for CHWs (Schneider et al., 2008). Respondents felt that community health leaders’ recommendations for appropriate incentives (C10) were distorted into meagre stipends. Keeping CHWs as volunteers may have serious implications for their motivation, retention and the quality of care they provide (F2).

The 2004 CHW framework suggests that the “government would provide grants to NGOs who would employ CHWs” (DoH, 2004). Three respondents identified partnership with civic organisations as positive but that the framework abdicates government financial responsibility and diminishes NGOs to stipend distributors (Schneider et al., 2008). In addition, the policy was seen to put insufficient emphasis on supervision, with directors deploring the fact that so few current budgets included adequate funds for supervision (F2). Quality of supervision, they suggested, is poor and is performed by inexperienced and overburdened staff.

## Discussion

This research contributes to filling a gap in the history of South African CHW programmes in the late apartheid period. These projects had similarities to the earlier community health initiatives of the 1940s. For example, as a consequence of the socio-political context, primarily outsiders, many of whom were white, middle-class professionals, started these projects. However, early and late apartheid projects evolved in different contexts. Due to a more liberal political leadership, the 1940s projects received government support as potential models for universal health care. In contrast, the late-apartheid projects studied were initiated within an era of heightened repression and segregation; were intertwined with the contemporaneous wider social aims of democracy and social justice, and did not generally receive backing from the state. Unfortunately, many late-apartheid programmes were poor at documenting the process and impact of their work, in part because of the repressive conditions. Projects of both apartheid periods, which were influenced by Community Oriented Primary Care, were much better at this, given the epidemiological focus of this model.

Our respondents argued that the strong socio-political motivations of the late apartheid period projects were mostly not carried through into the post-apartheid period. The current struggle to redress the economic, health and racial inequalities is not, it could be argued, fuelled by the same fervour for action, partly because the country is now a stable democracy (Friedman, 2002). In addition, the changing burden of disease (HIV, TB, chronic diseases) means that a national CHW programme would now have to incorporate significantly different needs (Oppenheimer & Bayer, 2007). However, a number of contemporary social movements, such as that to improve access to treatment for people living with HIV/AIDS, may have benefited from the leadership and experience of activists from the earlier projects (Ballard, Habib, Valodia, & Zuern, 2005).

Though these small-scale programmes were a product of their times, they have important lessons for current CHW programmes and policy within South Africa and potentially globally. It is suggested that the now predominant, single-purpose CHWs’ focus on clinical conditions fails to address the social determinants of health (Friedman, 2005). Reinvigorating the political nature of community healthcare by addressing social, economic and environmental issues, may have a greater impact in tackling ill health of the most disadvantaged.

Although interviewees claimed that dissatisfaction among CHWs with volunteering was minimal during apartheid, this may, in part, be a romanticisation of the past. In reality, many late apartheid programmes had very limited funding and repressive conditions often allowed few opportunities for local people other than voluntary involvement. Within their current work, CHW respondents indicated dissatisfaction with volunteering. Social changes in South Africa have created better local opportunities for people to contribute to health and seeking employment. Poorly- or un-remunerated involvement is perhaps no longer possible, and might be seen as exploitative (Lehmann, Friedman, & Sanders, 2004).

The current debate about the potential professionalisation of CHWs makes the ideal of ‘bridging a gap’ between the community and the health system more remote. For many younger workers, a CHW position is a stepping stone to a nursing career, not a long-term commitment to this cadre (Schneider et al., 2008). CHWs interviewed reported the community contact and trust experienced during the 1970s and 1980s as crucial to their work, although the degree of community participation was perhaps not as extensive and empowering as respondents claimed. Within the current model, CHWs risk becoming over-medicalised and no longer embedded in communities. Professionalisation may also blur the differences between nurses and CHWs, thereby contributing to power struggles between these two cadres. Furthermore, funding bodies’ centralised control on the remit and extent of these projects may curtail projects’ adequate community responsiveness.

Un-surprisingly, interviewees highlighted that good leadership and supervision, even though not always achieved, were essential to the success of programmes. Furthermore, community involvement and adequate financial capacity were seen as crucial for sustainability. Indeed, the ethos and funding flexibility of earlier programmes were seen to have led to community acceptance, CHW job satisfaction and health gain. Many problematic managerial issues then are also now further complicated by changes in the burden of disease and in user and provider expectations of health services. Many ongoing CHW programmes would probably benefit from stronger professional management and better integration within the district primary health system.

The issues identified in this historical analysis of CHW programmes are still recognised as important today but often remain poorly addressed, particularly in larger scale initiatives (Lehmann et al., 2004; Walt & Gilson, 1990). Given the renewed growth of CHW programmes within South Africa and globally, lessons learned from past programmes should play a stronger role in informing current policies.

## Figures and Tables

**Table 1:** Timeline of CHW projects and historical landmarks in South Africa.

Date	Event
1913	Natives Land Act (7.3% of South African land dedicated for Africans’ habitation)
1930s	Park Ross: Malaria assistants
1940s	Revival of African nationalism. African National Congress (ANC) rebuilt
1940	Pholela Health Unit founded by the Karks (Kwazulu)
1942–1944	National Health Services Commission
1945	Gluckman Report: intersectoral recommendations for comprehensive health service. Only health centres and IFCH established.
1948	Nationalist Party comes to power
1951	Bantu Authorities Act: forcible relocation of blacks to ‘homelands’
1951	Valley Trust established
1958	Karks’ exodus
1960	Sharpville massacre; ANC and Pan African Congress (PAC) banned.
1970	Chalumna/Newlands project started
1976	Soweto uprising: sparks nationwide uprisings
1976	Elim Care Groups started
1977	Steve Biko (leader of Black Consciousness Movement) tortured/dies in detention
1977	Public Health Act: dual/segregated healthcare
1979	Health Care Trust–VHW project started
1980	Valley Trust establishes CHW programme; SACLA clinic
1982	National Medical and Dental Association founded to address human rights
1985–1986	3 states of emergency: detentions/repression/assassinations
1986	SACLA clinic taken over by army
1986	Mamre Community Health Project and Rural Foundation Health Project started
1987	National Progressive Primary Health Care Network founded
1989	Relaxing of emergency laws
1990	ANC/PAC unbanned; progressive release of imprisoned leaders (including Mandela)
1991	Mamre/Zibonele projects start CHW programmes
1992–1994	Preliminary ANC health plan; national CHW workshops/conferences
1992	HCT– Brown’s Farm and Agincourt started (CHWs until mid-1990s)
1994	First democratic elections (Mandela elected); final health plan
1994–2003	Social/health policies promoting development and intersectoral collaboration (e.g. 1994: Reconstruction and Development Programme); Many late-apartheid CHW programmes close
2004	Community Health Policy Framework

**Table 2:** Details of projects.

Project	Comments	CHW characteristics	Current Status	Interviewees			
				CHW	C	D/N	F
**(PERI-)URBAN INITIATIVES**							
South African Christian Leadership Assembly Health Project	CHW programme in several peri-urban townships in Cape Town	Paid; generic and specialist (rehabilitation)	Running	1	3	2	1
Mamre Community Health Project	Coloured community. 3 components: research, CHW project, student teaching (academic)	Paid; specialist (youth, chronic illnesses, hypertension)	Closed	1	1		1
Health Care Trust – Brown’s Farm Project	Peri-urban township in Cape Town	Paid; generic	Closed				1
Zibonele	Peri-urban township in Cape Town (academic)	Paid; specialist (women, children)	Closed		1		1

**RURAL INITIATIVES**							
The Elim Care Group Project	Originally focussed on trachoma, then expanded to general health (Northern-Province)	Most volunteers; generic: (care-group volunteers, motivators, CHWs)	Running	8	1	1	
Chalumna and NewlandsVillage Health Worker Project	In the former Ciskei (Eastern Cape) Focus on nutrition, immunisation, TB	Stipend; generic	Closed				1
Health Care Trust – Village Health Worker Project	Xalanga district in the former Transkei (Eastern Cape)	Volunteers; generic	Closed				1
The Valley Trust Community Care Project	CHW project: part of Valley Trust – a large influential organisation, Kwazulu-Natal. Focus on health and ecology.	Volunteers then paid; generic	Running				1
The Rural Foundation Health Programme	Nationwide CHW programme for farm workers (started in Transvaal)	Paid by farmers; generic	CHW programme closed	2	4		1
National Progressive Primary Health Care Network	Umbrella organisation for progressive community health projects. Set up a CHW National Training Centre.		Closed		1	1	
Agincourt	Community health project with a focus on surveillance in Gazankulu (academic)	Paid research assistants	Running				1

C, coordinator; CHW, community health worker; D, doctor; N, nurse; F, founder.

**Table 3:** Comparison of past and current CHW programmes in South Africa.

Characteristics	1970–1990s programmes^b^	Current programmes
Supervision and training	Experimental but applied; done by experienced and inspiring people	Supervisors are of lower grades and less motivated/committed. Variable training quality^a^
Funding	International donors. More flexibility of allocation given to project by funders	Government-channelled funding, or charitable funds^a^ Rigid spending allowance, often determined by funders^b^
Remuneration	Variable, some CHWs well paid, many volunteers	Discontent with voluntary contributions^b^, low government stipend^a^
Scope	Started with a vertical issue, then extended to integrate larger health issues. PPHCN network established to coordinate projects.	Most are single-disease focused (e.g. HIV, TB) community-based organisations^a^
Relationship with community	Project more dependent on community and linked to activism. Community more participatory.	Different expectations since democracy (less dependence, more individualistic attitude)^b^
Relationship with health sector	Filling a large gap that health service was not providing. Mixed acceptance by health sector.	Poor coordination among projects/health sector, leading to competition and duplication of work^a^

a Based on (Friedman, 2005).

b Based on interviewees responses/contemporaneous literature.
